# Malondialdehyde Serum Levels in a Full Characterized Series of 430 Rheumatoid Arthritis Patients

**DOI:** 10.3390/jcm13030901

**Published:** 2024-02-04

**Authors:** Nayra Merino de Paz, Juan Carlos Quevedo-Abeledo, Fuensanta Gómez-Bernal, Antonia de Vera-González, Pedro Abreu-González, Candelaria Martín-González, Miguel Ángel González-Gay, Iván Ferraz-Amaro

**Affiliations:** 1 Division of Dermatology, Dermamedicin Clínicas, 38004 Santa Cruz de Tenerife, Spain; nayradepaz@hotmail.com; 2 Division of Rheumatology, Hospital Universitario Dr. Negrín, 35010 Las Palmas de Gran Canaria, Spain; jqueabe@gobiernodecanarias.org; 3 Division of Central Laboratory, Hospital Universitario de Canarias, 38320 Tenerife, Spain; fgomber@gobiernodecanarias.org (F.G.-B.); avergonq@gobiernodecanarias.org (A.d.V.-G.); 4 Unit of Physiology, Department of Basic Medical Sciences, University of La Laguna, 38200 Tenerife, Spain; pabreu@ull.edu.es; 5 Internal Medicine Department, Universidad de La Laguna, 38200 Tenerife, Spain; mmartgon@ull.edu.es; 6 Department of Medicine, University of Cantabria, 39005 Santander, Spain; 7 Division of Rheumatology, IIS-Fundación Jiménez Díaz, 28040 Madrid, Spain; 8 Division of Rheumatology, Hospital Universitario de Canarias, 38320 Tenerife, Spain

**Keywords:** rheumatoid arthritis, malondialdehyde serum levels, disease activity

## Abstract

**Background**. Oxidative stress has been involved in the pathogenesis of rheumatoid arthritis (RA). The serum malondialdehyde (MDA) level is a reliable biomarker of oxidative stress status. In the present work, we aimed to analyze how a comprehensive characterization of the disease characteristics in RA, including a lipid profile, insulin resistance, and subclinical atherosclerosis, relates to serum MDA levels. **Methods**. In a cross-sectional study that included 430 RA patients, serum MDA levels were evaluated. Multivariable analysis was performed to examine the relationship of MDA with disease activity scores and disease characteristics, including subclinical carotid atherosclerosis, a comprehensive lipid molecule profile, and indices of insulin resistance and beta cell function indices. **Results**. The erythrocyte sedimentation rate (ESR) showed a significant and positive relationship with MDA. However, this did not occur for other acute phase reactants such as C-reactive protein or interleukin-6. Although the DAS28-ESR score (Disease Activity Score in 28 joints) had a positive and significant association with MDA serum levels, other disease activity scores that do not use the erythrocyte sedimentation rate in their formula did not show a significant relationship with MDA. Other disease characteristics, such as disease duration and the existence of rheumatoid factor and antibodies against citrullinated protein, were not related to serum MDA levels. This also occurred for lipid profiles, insulin resistance indices, and subclinical carotid atherosclerosis, for which no associations with circulating MDA were found. **Conclusions**. The disease characteristics are not related to circulating MDA levels in patients with RA.

## 1. Introduction

Reactive oxygen species are generated as part of physiological processes and are typically produced at relatively modest levels in all cells and tissues, and the primary internal source of these species is the electron transport chain within the mitochondria [1]. Nitric oxide, nitrogen dioxide, and peroxynitrite represent the most important ones. These chemical species possess one or more unpaired electrons in their outermost orbital shell, earning them the label of free radicals. They exhibit instability, high reactivity, and a short lifespan. Free radicals have the ability to attract electrons from other compounds to achieve stability, causing the target molecule to lose its electron and transform into a free radical, initiating a cascade of chain reactions [2]. Under normal physiological conditions, reactive oxygen species are essential for maintaining cellular redox balance and contribute to processes like cell signaling, differentiation, proliferation, growth, apoptosis, cytoskeletal regulation, and phagocytosis [3]. However, when reactive oxygen species levels exceed physiological norms, they can inflict damage upon cellular components, including cell membrane lipids, proteins, and nucleic acids [4]. If a particular situation leads to an imbalance favoring oxidants over antioxidants, it disrupts redox signaling and results in cellular damage and/or loss of control. The cellular condition referred to as oxidative stress can occur due to an overabundance of oxidants, a lack of antioxidants, or a combination of both factors [5].

Biomarkers of oxidative stress can be categorized into two groups: molecules that undergo modifications due to interactions with reactive oxygen species in the cellular environment and molecules from the antioxidant system that undergo changes in response to heightened redox stress [6]. DNA, lipids (comprising phospholipids), proteins, and carbohydrates are all molecules that can be negatively impacted by an excessive presence of reactive oxygen species within the body. Notably, lipids are particularly vulnerable to oxidation due to their molecular structure, which contains a significant number of reactive double bonds [7]. Malondialdehyde (MDA) is among the most widely recognized indicators of lipid peroxidation. MDA is generated in vivo through the peroxidation of polyunsaturated fatty acids and is typically measured using plasma samples. The prevalent technique for this assessment is a colorimetric assay that relies on the reaction between MDA and thiobarbituric acid [8]. MDA is a commonly employed biomarker for oxidative stress assessment [9]. Furthermore, there is a hypothesis suggesting that MDA may interfere with the interaction between OxLDL cholesterol and macrophages, potentially contributing to the progression of atherosclerosis [10,11].

Rheumatoid arthritis (RA) is the predominant chronic type of inflammatory arthritis. The primary pathology of RA originates within the synovium of diarthrodial joints, although non-articular organs and tissues may also experience involvement. In this sense, involvement of the musculoskeletal system other than joints (e.g., bone and muscle) and of non-articular organs (e.g., skin, eye, lungs, heart, and others) occurs in approximately 40 percent of patients with RA over the course of the disease [12]. The pathogenesis of RA is complex, with multiple genetic, environmental, immunologic, and other factors contributing to the development and expression of the disease [13].

In the past few decades, it has been proposed that oxidative stress plays a role in the advancement of rheumatoid arthritis (RA) by causing damage to DNA, lipids, and proteins, which subsequently leads to inflammation in the synovial tissue [14]. For example, circulating lymphocytes from patients with RA recognize oxidatively modified IgG in vitro by initiating a proliferative response and secreting interleukin 2 [15]. Reactive oxygen and nitric oxide products secreted by inflammatory cells generate covalent crosslinked IgG aggregates with biologic properties of true immune complexes in RA [16]. Furthermore, existing evidence shows the potential benefits of antioxidant stress therapy for oxidative stress levels in RA, improving the clinical and inflammatory indices of the disease [17]. In this sense, antioxidant therapies like N-acetylcysteine [18], coenzyme Q10 [19], probiotics [20], vitamins A, E, and C [21], ozone [22], or selenium [23] have shown potential benefits in RA. However, more large samples and quality randomized clinical trials are needed to provide high-quality evidence. Remarkably, MDA has been implicated in RA. In this regard, a new antibody system directed at MDA adducts created by oxidative stress has been identified in patients with RA [24]. These antibodies are strongly associated with the presence of anticitrullinated protein antibodies. Sixteen studies that assessed MDA in RA serum or synovial fluid were assessed in a systematic review [25]. Eight of the ten that measured MDA serum levels described a statistically significant increase in MDA blood levels in RA patients. However, a significant difference in MDA blood concentration between RA and control patients was not reported in another two [25]. Furthermore, disagreement has been described regarding the relationship of MDA with characteristics of the disease such as its clinical or inflammatory activity [26,27,28]. Furthermore, these studies generally lack multivariable analysis, or the number of subjects recruited was relatively small.

Our objective in this work was to study the relationship between the characteristics of the disease and serum levels of MDA. To do this, we characterized a large series of patients with RA. In addition to disease characteristics, information on dyslipidemia, subclinical atherosclerosis, and insulin resistance was collected. Multivariable regression analysis was planned to analyze possible associations.

## 2. Materials and Methods

### 2.1. Study Participants

This was a cross-sectional study that encompassed 430 consecutively enrolled RA patients, all of whom were 18 years of age or older and met the 2010 ACR/EULAR classification criteria [29]. They were diagnosed by rheumatologists and received regular follow-up care at rheumatology outpatient clinics. Patients were required to have a minimum RA disease duration of ≥1 year. Patients taking prednisone or an equivalent dose ≤10 mg/day, which is commonly used in RA management, were allowed to participate in the study. Exclusion criteria encompassed a prior history of angina, stroke, myocardial infarction, a glomerular filtration rate below 60 mL/min/1.73 m^2^, a history of cancer, or any other enduring ailments such as heart or respiratory diseases, hypothyroidism, nephrotic syndrome, as well as indications of ongoing infection. The study protocol was sanctioned by the Institutional Review Committees at Hospital Universitario de Canarias and Hospital Universitario Doctor Negrín (both located in Spain), with all participants providing informed written consent (approval no. 2019-452-1). Adherence to pertinent guidelines and regulations was ensured, adhering to the principles outlined in the Declaration of Helsinki.

### 2.2. Data Collection and Laboratory Assessments

Participants in the study filled out a custom questionnaire concerning cardiovascular risk factors and medication usage, and they also underwent a physical examination. Standardized protocols were adhered to for the assessment of body mass index (BMI), a metric computed by weight in kilograms divided by the square of height in meters. Furthermore, measurements were taken for abdominal circumference and systolic and diastolic blood pressure. Data regarding smoking habits, diabetes, and hypertension status were acquired through a questionnaire. Specific diagnoses and medication particulars were retrieved by reviewing medical records. Disease activity in patients with RA was assessed using the Clinical Disease Activity Index (CDAI) [30], the Simple Disease Activity Index (SDAI) [31], and the Disease Activity Score (DAS28) in 28 joints [32]. HDL-cholesterol, total cholesterol, and triglycerides were assessed using an enzymatic colorimetric assay. LDL-cholesterol was calculated through the Friedewald formula. The erythrocyte sedimentation rate (ESR) and high-sensitivity C-reactive protein (CRP) were measured using standard techniques. To assess insulin resistance (IR), the homeostatic model assessment (HOMA) method was employed. In brief, the HOMA model allowed for the estimation of insulin sensitivity (%S) and β-cell function (%B) based on fasting serum insulin, C-peptide, and glucose concentrations. In this study, we utilized HOMA2, an updated computer-based version of the HOMA model [33]. Human interleukin-6 was assessed by electrochemiluminescence immunoassay method (Roche Diagnostics, Indianapolis, IN, USA).

The Thiobarbituric Acid Reactive Substance (TBARS) assay is a technique utilized for the detection of lipid oxidation. This assay is specifically designed to quantify MDA, which is one of the final products formed during the degradation of lipid peroxidation substances. The levels of MDA in the serum were assessed using a modified approach based on the method originally outlined by Kikugaw et al. [34]. To perform the assay, a 0.2 mL volume of the sample was combined with 0.2 mL of 0.2 M H_3_PO_4_ (Merck Life Science, Madrid, Spain). The color reaction was initiated by adding 25 µL of a 0.11 M thiobarbituric acid (TBA, Sigma-Aldrich, Madrid, Spain) solution. The mixture was then heated at 90 °C for 50 min using a heating block. After cooling, the TBARS (resulting in a pink complex color) were removed by adding 0.4 mL of n-butanol (Sigma-Aldrich, Madrid, Spain). Centrifugation at 6000× *g* for 10 min allowed for the split of the butanolic phase. Each sample was relocated to a 96-well plate and read at 535 nm by means of a microplate spectrophotometer reader (Spectra MAX-190, Molecular Devices, Sunnyvale, CA, USA). A calibration curve was arranged using authentic MDA standards (Merck Life Science, Madrid, Spain). The assay’s detection limit was established at 0.079 nmol/mL. Intra-assay and inter-assay coefficients of variation were calculated at 1.82% and 4.01%, respectively. The serum concentration of MDA was quantified in nmol per ml. To diminish potential interferences from compounds that react or absorb at 532 nm, each sample was accompanied by a blank tube (sample without the TBA reagent), and the absorbance of the blank tube was subtracted from each sample measurement [35]. Additionally, the use of butanol as the extracting agent for the TBARS complex helped to lessen many of these interferences [36].

### 2.3. Cardiovascular Risk and Carotid Ultrasound Assessments

Cardiovascular risk score 2 [SCORE2] was processed according to the 2021 European Society of Cardiology Guidelines on cardiovascular disease prevention in clinical practice for the Spanish population, which is considered a low-risk region [37]. The SCORE2 risk categories are categorized as low to moderate, high, and very high, depending on distinct age groups, which are defined as follows: those under 50, those between 50 and 69, and those aged 70 or older. SCORE2 is a tool that calculates an individual’s 10-year risk of cardiovascular disease events that could be fatal or non-fatal, specifically designed for individuals aged 40 to 69 years. In the case of healthy individuals aged 70 or older, the SCORE2-OP (older persons) algorithm is employed to estimate both 5-year and 10-year risks of fatal and non-fatal cardiovascular disease events.

Carotid ultrasound was employed to evaluate the carotid intima-media thickness (cIMT) in the common carotid artery and to identify focal plaques in the extracranial carotid arteries [38]. A commercially available ultrasound scanner, the EsaoteMylab 70 (Genoa, Italy), equipped with a 7–12 MHz linear transducer and automated software-guided radiofrequency technology, Quality Intima Media Thickness in real-time (QIMT, Esaote, Maastricht, Holland), was utilized for this purpose. As previously described [38], in accordance with the Mannheim consensus, plaque criteria in the accessible extracranial carotid arteries (comprising the common carotid artery, bulb, and internal carotid artery) were defined as follows: a focal protrusion within the lumen measuring at least cIMT > 1.5 mm; a protrusion at least 50% larger than the surrounding cIMT; or an arterial lumen encroachment > 0.5 mm [39].

### 2.4. Statistical Analysis

The clinical characteristics and demographics of RA patients were described as a mean (standard deviation) or percentages for categorical variables. In the case of non-normally distributed continuous variables, data were presented as the median and interquartile range (IQR). To examine the relationship between disease-related data and MDA while accounting for confounding factors, a multivariable linear regression analysis was planned. Confounding variables would be selected from traditional cardiovascular risk factors or demographics if they exhibited a *p*-value below 0.20 in the univariable association with MDA. All analyses were conducted at a two-sided significance level of 5% using Stata software, version 17/SE (StataCorp, College Station, TX, USA). *p*-values less than 0.05 were considered statistically significant.

## 3. Results

### 3.1. Demographic and Disease-Related Data

A total of 430 rheumatoid arthritis (RA) patients participated in this study. The demographic and disease-related characteristics of the participants are summarized in Table 1. The mean age of the participants was 55 ± 10 years, with 81% being women. The patients had an average body mass index (BMI) of 29 ± 15 kg/m^2^ and an abdominal circumference of 97 ± 13 cm. Common classical cardiovascular risk factors were observed: 22% were smoking, 13% had type 2 diabetes, 32% were cataloged as obese (BMI equal to or greater than 30 kg/m^2^), and 34% had hypertension. Furthermore, 32% of the patients were under statins at the time of recruitment.

In this group of RA patients, the median disease duration was 8 years (IQR: 4–15 years). At the time of the study, CRP levels were 2.7 (IQR: 1.3–6.1) mg/L, and ESR levels were 18 (IQR: 7–32) mm/1st hour. Sixty-five percent tested positive for anti-citrullinated protein antibodies and 72% of the patients were positive for rheumatoid factor. Disease activity, as measured by DAS28-ESR, averaged 3.1 ± 1.4. Thirty-six percent of the patients were receiving prednisone treatment, and 87% were taking at least one form of conventional disease-modifying antirheumatic drug (DMARD), with methotrexate being the most prescribed (73%). Nineteen percent of the patients were undergoing anti-tumor necrosis factor therapies. Additional details regarding treatment frequencies and historical disease-related data can be found in Table 1.

### 3.2. Relation between Demographic and Disease-Related Data and MDA Serum Levels

The relationship of demographic data, cardiovascular risk factors, and disease data to MDA is shown in Table 2. Age, sex, and anthropometric data did not show significant associations with MDA serum levels. This was also the case for cardiovascular risk factors, including statin use. Regarding disease data, neither disease duration nor the presence of rheumatoid factor or ACPA were associated with MDA values. Although ESR showed a significant and positive relationship with MDA, this was not the case for other acute phase reactants such as CRP or IL-6.

With respect to disease activity, the DAS28-ESR score showed a positive and significant association with MDA serum levels. However, other disease activity scores that do not use ESR in their formula did not show a significant relationship with MDA. Furthermore, when disease activity scores were categorized as in remission and as moderate, high, and very high disease activity no relationship with MDA was found.

Regarding the use of therapies, only the NSAID intake showed a significant relationship with MDA, in this case negative (Table 2). However, the significant associations in this analysis were not adjusted for covariates because none of the demographics or cardiovascular risk factors disclosed a *p*-value less than 0.20. Moreover, when this analysis was performed separately in patients positive and negative for rheumatoid factor and ACPA, the same associations were found.

### 3.3. Relationship of Cardiovascular Risk Score, Carotid Atherosclerosis, and Lipid Profile and Insulin Resistance Indices to MDA Serum Levels

The mean cIMT had a value of 696 ± 131 microns, and 42% of the patients had carotid plaques. The median SCORE2 value was 3.7 (IQR 1.8–5.9) %). Furthermore, according to SCORE2, 62% were in the low or moderate cardiovascular risk category, and 25 and 13% of the subjects were in the high and very high cardiovascular risk class, respectively (Table 3). 

The full lipid profile and insulin resistance indices (calculated only for non-diabetic patients and if glucose was ≤110 mg/dL) are described in Table 3. Remarkably, no relationship was found between all these cardiovascular risk factors and the SCORE2 cardiovascular risk algorithm with serum MDA levels (Table 3). In this evaluation, multivariable analysis was not performed since no significant relationships were found. Additionally, we performed the same analysis of this table in only patients with a BMI ≥ 25 kg/m^2^. In this group of patients, neither relation was found between MDA and metabolic parameters.

## 4. Discussion

This study includes the largest series of RA patients with data on serum MDA levels. This series was fully characterized, including information on demographic variables, disease-related features, comorbidity of other organs, and accompanying cardiovascular disease. According to our findings, circulating MDA is not related to disease characteristics. Since MDA was only related to ESR but not to other inflammatory markers, we consider that MDA cannot be used as a reliable marker of disease activity in RA. Furthermore, we could not find a relationship between MDA and dyslipidemia or insulin resistance in RA. Therefore, serum MDA level does not appear to be useful as a biomarker of disease activity or metabolic syndrome in patients with RA.

Several studies evaluated serum MDA levels in RA. However, they included a small number of subjects and generally lacked multivariable adjustment. Furthermore, these studies generally did not analyze the relationship of MDA with disease characteristics. In this regard, increased MDA serum levels were described in a study that compared 20 RA patients with 20 controls [40]. Serum MDA levels were reported to be higher in 31 patients with active RA compared to 12 patients with inactive disease [41]. Elevated serum MDA levels were also found in 55 RA patients compared to 25 controls, but in this study, no differences in circulating MDA levels were found between active RA patients and inactive RA patients [42]. A significant correlation between serum MDA and several inflammatory chemokines was described in a study that included 30 patients with RA [43]. In contrast, in a study comparing 36 patients with RA and 36 controls, circulating MDA levels were higher in those with RA but MDA was not associated with CRP [44]. In a series of 36 patients, MDA levels in synovial fluid showed a correlation with the disease activity score [28]. Another report on 49 RA patients described a positive correlation between anticitrullinated peptide antibody levels and synovial MDA [27]. As observed in a study that described no differences in serum MDA levels between 35 patients with RA and 39 controls, serum MDA levels did not show a correlation with CRP levels [45]. In our series of patients with RA, we did not find a relationship between MDA serum levels and disease characteristics such as disease duration, activity scores, and the presence of rheumatoid factor or anti-citrullinated peptide antibody. Apart from ESR, we could not observe a relationship between serum MDA levels and other markers of inflammation such as CRP or IL-6. At this point, we believe it is important to note that, unlike all the studies mentioned above, our work included a much larger number of patients, and multiple disease characteristics related to non-articular comorbidity were also evaluated.

Patients with RA often exhibit metabolic abnormalities such as dyslipidemia or insulin resistance that are included in the definition of metabolic syndrome [46]. They have a state of inflammatory dyslipidemia that is associated with a decrease in certain lipid molecules [47]. However, the relationship between MDA, a biomarker of lipid peroxidation, and the lipid pattern has barely been studied in patients with RA. In this regard, Mishra et al. did not observe a correlation between MDA and total cholesterol, LDL-cholesterol, and HDL-cholesterol in 36 patients with RA [44]. Consistent with these findings, we observed no associations between MDA and lipid-related molecules in our larger series. Therefore, according to our data, inflammatory dyslipidemia in RA does not appear to be related to serum MDA levels. This would also be the case for the insulin resistance frequently seen in RA patients [48,49]. In this sense, although it has been described that type 2 diabetes mellitus is related to an increase in plasma lipid peroxidation expressed as malondialdehyde [50,51], and the frequency of diabetes is increased in patients with RA, MDA does not seem to be responsible for the alteration of glucose metabolism that usually accompanies RA.

MDA has also been associated with the risk of mortality in patients with chronic heart failure and is related to traditional cardiovascular risk factors [52]. However, we did not find a relationship between subclinical carotid atherosclerosis and MDA in RA. 

With regard to disease duration and the biomarkers of the disease, a study of our group showed a significant negative relationship with disease duration but a positive relationship with anti-nucleosome antibodies in 284 patients with systemic lupus erythematosus [53]. Moreover, the musculoskeletal and cutaneous accrual damage over time in these patients with systemic lupus erythematosus was related to higher serum MDA values. Similarly, complement activation was associated with higher serum levels of MDA [53]. However, based on the results of the current work, this does not appear to be the case for RA. Similarly, MDA has been described as not related to disease characteristics in patients with systemic sclerosis [54]. It is possible that different pathological mechanisms leading to MDA expression in RA and other autoimmune diseases may explain this fact.

Antioxidants have been shown in some studies to have protective effects against tissue damage and may lead to clinical improvement in these patients [55]. This is believed to be the consequence of the antioxidant compounds reducing inflammation by exertion their effects on the transcription factor of NF-κB in RA patients. In one recent study, coenzyme Q10, a fat-soluble antioxidant, showed a decreasing effect on serum MDA and the proinflammatory cytokine of TNF-α in RA [56]. However, the literature still lacks studies regarding the long-term effect of antioxidant supplementation in RA.

Our study has the advantage of having included a large number of well-characterized patients. This would have allowed multivariable analysis to be carried out. However, it was not necessary since no significant associations were found. Furthermore, we acknowledge the limitations of this study. In this sense, we did not recruit controls. However, our intention was not to study the differences between patients with RA and healthy controls in MDA values but rather to evaluate their relationship with the characteristics of the disease. Furthermore, the cross-sectional design of our work prevents inferring causality. Prospective cohort studies are also needed to assess how MDA presents in the naïve stage, how it evolves over time after therapies, or its relationship to relapses in RA patients. In addition, oxidative stress can affect other substances such as DNA, proteins, and urate, among others. In addition, there are several antioxidant regulators, such as superoxide dismutase and glutathione peroxidase, which were not evaluated in our study. For this reason, we cannot rule out that other biomarkers of oxidative stress may be related to disease characteristics in RA. Furthermore, we acknowledge that we have not recorded the use of hormone replacement therapy in our patients. However, this practice is unusual in our country. For this reason, we consider that this has not affected the results of our study. Also, 80% of the recruited patients were women. This is in line with the fact that RA predominantly affects women. For this reason, our results in men must be taken with caution since the number of male patients was lower. Likewise, methotrexate was used in three-quarters of the patients, and most of the patients were in the remission or low disease activity categories. However, we believe that this has not influenced our results because patients with high activity did not show higher MDA levels compared to those who had low activity or were in remission. Similarly, since this information was not recorded, we cannot conclude what effects vitamin D, or environmental factors such as diet, may have had on MDA in RA patients.

## 5. Conclusions

In conclusion, although MDA adducts, or antibodies directed against them, have been related to the etiopathogenesis of RA, serum MDA levels are not associated with disease expression. Therefore, it may be the ability of MDA to generate autoimmunity, and not its serum levels, that is how this oxidative stress biomarker is related to RA. The measurement of oxidative stress through MDA in RA patients does not serve as a biomarker for monitoring disease activity or severity in RA.

## Figures and Tables

**Table 1:** Demographics, cardiovascular risk factors, and disease-related data in patients with RA.

	Rheumatoid Arthritis
	(*n* = 430)
Age, years	55 ± 10
Female, *n* (%)	350 (81)
Hip circumference, cm	106 ± 11
Abdominal circumference, cm	97 ± 13
Waist-to-hip circumference ratio	0.92 ± 0.08
BMI, kg/m^2^	29 ± 15
Cardiovascular risk factors	
Current smoker	93 (22)
Obesity	137 (32)
Dyslipidemia	200 (47)
Diabetes Mellitus	54 (13)
Hypertension	148 (34)
Statins, *n* (%)	139 (32)
Disease-related data	
Disease duration, years	8 (4–15)
CRP, mg/L	2.7 (1.3–6.1)
ESR, mm/1st hour	18 (7–32)
IL-6, pg/mL	5.0 (3.2–8.6)
ACPA, *n* (%)	253 (65)
Rheumatoid factor, *n* (%)	303 (72)
SDAI	12 (7–19)
CDAI	8 (4–14)
DAS28-ESR	3.13 ± 1.35
DAS28-CRP	2.73 ± 1.08
Erosions, *n* (%)	166 (43)
History of extraarticular manifestations, *n* (%)	38 (10)
Current drugs, *n* (%)	
Prednisone	155 (36)
Prednisone doses, mg/day	5 (3–5)
DMARDs	373 (87)
NSAIDs	194 (45)
Hydroxychloroquine	45 (18)
Salazopyrin	28 (7)
Methotrexate	316 (73)
Leflunomide	94 (22)
Anti-TNF therapy	83 (19)
Tocilizumab	23 (5)
Rituximab	7 (2)
Abatacept	12 (3)
JAK inhibitors	20 (5)
Baricitinib	6 (1)
Tofacitinib	11 (3)

The data are presented as either mean ± SD or median (IQR) when the data deviate from a normal distribution. CRP stands for C-reactive protein. NSAID refers to nonsteroidal anti-inflammatory drugs, DMARD stands for disease-modifying antirheumatic drug, and TNF represents tumor necrosis factor. Additionally, the terms ESR (erythrocyte sedimentation rate), BMI (body mass index), DAS28 (Disease Activity Score in 28 joints), ACPA (Anti-citrullinated protein antibodies), CDAI (Clinical Disease Activity Index), and SDAI (Simple Disease Activity Index) are used in the context of these data.

**Table 2:** The relation between demographic and disease-related data and MDA serum levels in patients with RA.

	MDA nmol/mL	
	Beta Coef. (95%CI)	*p*
Age, years	−0.002 (−0.02–0.02)	0.85
Female, *n* (%)	−0.09 (−0.5–0.4)	0.69
Hip circumference, cm	0.00003 (−0.02–0.02)	0.99
Abdominal circumference, cm	−0.0007 (−0.01–0.01)	0.92
Waist-to-hip circumference ratio	−0.2 (−2–2)	0.88
BMI, kg/m^2^	−0.002 (−0.01–0.007)	0.63
Cardiovascular risk factors and data		
Current smoker	0.06 (−0.3–0.5)	0.76
Obesity	−0.1 (−0.5–0.2)	0.45
Dyslipidemia	−0.09 (−0.4–0.2)	0.60
Diabetes Mellitus	−0.3 (−0.8–0.2)	0.20
Hypertension	−0.1 (−0.5–0.2)	0.44
Statins, *n* (%)	−0.1 (−0.5–0.3)	0.60
Disease-related data		
Disease duration, years	−0.006 (−0.02–0.01)	0.49
CRP, mg/L	−0.003 (−0.02–0.009)	0.61
ESR, mm/1st hour	**0.01 (0.003–0.02)**	**0.010**
IL-6, pg/mL	0.01 (−0.0002–0.02)	0.055
ACPA, *n* (%)	0.1 (−0.2–0.5)	0.45
Rheumatoid factor, *n* (%)	0.04 (−0.3–0.4)	0.84
DAS28-CRP	0.1 (−0.05–0.3)	0.19
DAS28-ESR	**0.1 (0.008–0.3)**	**0.038**
SDAI	0.0003 (−0.01–0.01)	0.96
CDAI	0.01 (−0.01–0.03)	0.30
Erosions, *n* (%)	−0.2 (−0.5–0.2)	0.33
History of extraarticular manifestations, *n* (%)	0.1 (−0.5–0.7)	0.76
Current drugs, *n* (%)		
Prednisone	0.2 (−0.1–0.6)	0.21
Prednisone doses, mg/day	0.02 (−0.08–0.1)	0.70
DMARDs	−0.2 (−0.7–0.3)	0.51
NSAIDs	**−0.4 (−0.8–(−0.09))**	**0.013**
Hydroxychloroquine	0.1 (−0.5–0.7)	0.77
Salazopyrin	0.08 (−0.7–0.9)	0.85
Methotrexate	−0.1 (−0.5–0.3)	0.55
Leflunomide	−0.03 (−0.5–0.4)	0.87
Anti-TNF therapy	−0.3 (−0.8–0.1)	0.15
Tocilizumab	−0.1 (−0.9–0.7)	0.75
Rituximab	−0.4 (−2–0.8)	0.47
Abatacept	0.1 (−1–1)	0.79
JAK inhibitors	−0.4 (−1–0.4)	0.37
Baricitinib	−0.4 (−2–1)	0.60
Tofacitinib	−0.2 (−1–1)	0.68

In this analysis, MDA serves as the outcome variable. MDA stands for Malondialdehyde, while other variables include CRP (C-reactive protein), NSAID (Nonsteroidal anti-inflammatory drugs), DMARD (disease-modifying antirheumatic drug), TNF (tumor necrosis factor), ESR (erythrocyte sedimentation rate), BMI (body mass index), DAS28 (Disease Activity Score in 28 joints), ACPA (Anti-citrullinated protein antibodies), CDAI (Clinical Disease Activity Index), and SDAI (Simple Disease Activity Index). Significant *p*-values are indicated in bold.

**Table 3:** The relationship of the cardiovascular risk SCORE2 algorithm, carotid atherosclerosis, and the lipid profile and insulin resistance indices to MDA serum levels.

		MDA nmol/mL	
		Beta Coef. (95% CI)	*p*
SCORE2			
SCORE2, %	3.7 (1.8–5.9)	0.006 (−0.04–0.05)	0.78
Low or moderate risk	265 (62)	ref.	
High risk	108 (25)	0.3 (−0.1–0.7)	0.21
Very high risk	57 (13)	0.06 (−0.5–0.6)	0.82
Carotid ultrasound			
cIMT, microns	696 ± 131	0.5 (−1–2)	0.47
Carotid plaque, *n* (%)	180 (42)	0.03 (−0.3–0.4)	0.84
Lipid profile			
Total cholesterol, mg/dL	205 ± 38	0.002 (−0.002–0.007)	0.40
Triglycerides, mg/dL	147 ± 86	0.00005 (−0.0003–0.0002)	0.68
Apolipoprotein A1, mg/dL	173 ± 31	−0.0006 (−0.006–0.005)	0.84
Apolipoprotein B, mg/dL	106 ± 26	−0.0005 (−0.007–0.006)	0.89
Apo B:Apo A1 ratio	0.63 ± 0.24	−0.04 (−1–1)	0.94
Apolipoprotein C3, mg/dL	4.8 (2.2–8.7)	−0.002 (−0.03–0.02)	0.83
LDL-cholesterol, mg/dL	120 ± 34	0.0005 (−0.0008–0.002)	0.43
HDL-cholesterol, mg/dL	57 ± 15	−0.007 (−0.02–0.004)	0.19
LDL:HDL cholesterol ratio	2.27 ± 0.93	0.05 (−0.03–0.1)	0.23
Non-HDL cholesterol, mg/dL	149 ± 39	0.003 (−0.001–0.008)	0.17
Lipoprotein (a), mg/dL	34 (11–107)	−0.001 (−0.004–0.0008)	0.21
Insulin resistance values *			
Glucose, mg/dL	95 ± 24	−0.00009 (−0.009–0.008)	0.98
C-peptide, ng/mL	2.5 (1.6–4.0)	0.02 (−0.07–0.1)	0.70
Insulin, µU/mL	8.6 (5.5–15.1)	−0.002 (−0.008–0.005)	0.62
HOMA2-IR	1.09 (0.7–2.0)	0.02 (−0.1–0.2)	0.76
HOMA2-S%	92 (51–142)	0.0006 (−0.004–0.005)	0.80
HOMA2-B%-C-peptide	162 ± 77	−0.0003 (−0.004–0.003)	0.87

The data are presented as either mean ± SD or median (IQR) when the data do not follow a normal distribution. LDL represents low-density lipoprotein, HDL stands for high-density lipoprotein, and cIMT is an abbreviation for the carotid intima-media thickness. * The analysis focuses on the relationship between insulin resistance indices and MDA, but this calculation is only completed for non-diabetic patients with glucose levels below 110 mg/dL. MDA, which stands for Malondialdehyde, serves as the dependent variable in this analysis. SCORE is Systematic Coronary Risk Evaluation and HOMA is homeostatic model assessment.

## Data Availability

The data sets used and/or analyzed in the present study are available from the corresponding author upon request.
